# Breeding Targets to Improve Biomass Quality in Miscanthus

**DOI:** 10.3390/molecules26020254

**Published:** 2021-01-06

**Authors:** Kasper van der Cruijsen, Mohamad Al Hassan, Gijs van Erven, Oene Dolstra, Luisa M. Trindade

**Affiliations:** 1 Laboratory of Plant Breeding, Wageningen University & Research, Droevendaalsesteeg 1, 6708 PB Wageningen, The Netherlands; kasper.vandercruijsen@wur.nl (K.v.d.C.); mohamed.alhassan@wur.nl (M.A.H.); oene.dolstra@wur.nl (O.D.); 2 Wageningen Food and Biobased Research, Bornse Weilanden 9, 6708 WG Wageningen, The Netherlands; gijs.vanerven@wur.nl; 3 Laboratory of Food Chemistry, Wageningen University & Research, Bornse Weilanden 9, 6708 WG Wageningen, The Netherlands

**Keywords:** miscanthus, lignocellulosic biomass, saccharification, cell wall, pretreatment, breeding, cellulose, hemicellulose, lignin, biomass quality

## Abstract

Lignocellulosic crops are attractive bioresources for energy and chemicals production within a sustainable, carbon circular society. Miscanthus is one of the perennial grasses that exhibits great potential as a dedicated feedstock for conversion to biobased products in integrated biorefineries. The current biorefinery strategies are primarily focused on polysaccharide valorization and require severe pretreatments to overcome the lignin barrier. The need for such pretreatments represents an economic burden and impacts the overall sustainability of the biorefinery. Hence, increasing its efficiency has been a topic of great interest. Inversely, though pretreatment will remain an essential step, there is room to reduce its severity by optimizing the biomass composition rendering it more exploitable. Extensive studies have examined the miscanthus cell wall structures in great detail, and pinpointed those components that affect biomass digestibility under various pretreatments. Although lignin content has been identified as the most important factor limiting cell wall deconstruction, the effect of polysaccharides and interaction between the different constituents play an important role as well. The natural variation that is available within different miscanthus species and increased understanding of biosynthetic cell wall pathways have specified the potential to create novel accessions with improved digestibility through breeding or genetic modification. This review discusses the contribution of the main cell wall components on biomass degradation in relation to hydrothermal, dilute acid and alkaline pretreatments. Furthermore, traits worth advancing through breeding will be discussed in light of past, present and future breeding efforts.

## 1. Introduction

Increased carbon dioxide levels are the foremost cause of anthropogenic climate change leading to global warming [1]. The goal of keeping the global average temperature well below a 2 °C increase from the pre-industrial levels has been set in the Paris Agreement, in order to halt global warming. To reach this goal, it is important to develop methods or set measures to reduce CO_2_ levels [2,3]. The majority of CO_2_ emissions are associated with the production of energy and synthetic polymers from fossil resources as oil, coal and natural gas [4,5]. Therefore, there is an increasing demand for less polluting alternatives for the production of energy and chemicals. While there are several renewable sources for energy production, such as solar and wind power, only biomass can serve both purposes. Although skepticism exists about the capacity of biomass usage regarding mitigation of CO_2_ emissions [6], thorough assessments have shown that biomass has the potential to offset greenhouse gas emissions when properly used, and could be an integral part of a wider strategy in order to meet global climate goals [7,8]. Transition towards a biobased economy requires change across the whole production chain, with particular importance for advancements regarding efficient biomass production, conversion into different products and utilization [9,10].

Biomass itself is an attractive renewable energy source due to its potential to be carbon neutral and its global abundance. Carbon neutrality depends on the basic principle that the amount of CO_2_ released upon combustion and or conversion is equal to the amount fixed by the crop during its lifetime [11]. As such, replacement of traditional fossil energy sources that heavily contribute to elevated levels of CO_2_ with biomass energy can alleviate the associated effect on global warming [12]. Additionally, biomass provides an essentially unlimited source of natural and renewable building blocks for utilization as alternatives to oil-derived chemicals and materials [13].

Lignocellulosic biomass, retrieved from agricultural and forest side-streams or dedicated feedstocks is expected to become an essential resource for the production of energy, chemicals and materials in the near future. Dedicated biomass crops are needed next to agricultural and forest streams, because the contribution of the latter alone would be insufficient for meeting the energy demands [14,15,16,17]. Perennial C4 grasses have been considered as especially promising feedstocks due to their more efficient photosynthetic capacity relative to C3 plants. This is in most cases associated with higher biomass yield potential and increased nitrogen and water use efficiencies [18]. Moreover, their perennial nature also contributes to higher nutrient use efficiency in comparison to annual crops [19]. These features enable perennial grasses, like switchgrass and miscanthus, to achieve substantial yields even when cultivated on marginal and degraded lands [20,21,22]. Limiting cultivation to marginal lands avoids competition for arable land with food crops and will therefore not present a threat to food prices and security or induce land use change; both were points of concern and criticism accompanying the use of edible parts of food crops for the production of first generation biofuels [21,23,24]. Additionally, due to the perennial grasses’ capacity to sequestrate CO_2_ [25,26] and thereby tilt the carbon balance more favorably, it can be assumed that the detrimental effects of large scale use of forestry biomass on net CO_2_ emissions [27,28] would not apply to these crops. Therefore, cultivation of perennial grasses can be seen as a sustainable alternative without any obvious negative societal impacts. From the available candidate biomass crops, miscanthus is seen as one of the most promising as it is able to utilize external resources even more efficiently than other C4 grasses [19,29].

### 1.1. Miscanthus for Industrial Use: Advantages, Challenges and Applications

Miscanthus is a genus of rhizomatous perennial grasses originating from Eastern Asia, which comprises around 12 different species [30,31]. The species have been adapted to a broad range of different climate conditions and hold substantial amounts of genetic diversity for key traits [32,33]. Interest in miscanthus has been, for a large part, due to excellent biomass yields that are provided on a yearly basis and could be achieved without the need of additional irrigation in Northern Europe [34]. Such yield potential is achieved due to its ability to maintain photosynthetic capacity at moderate temperatures [35,36]. Furthermore, low input requirements due to its high levels of water [29,37,38] and nutrient use efficiency [39,40] are also highly favorable characteristics of miscanthus species.

Initially, most research has focused on *M. x giganteus*, an interspecific sterile hybrid between *M. sinensis* and *M. sacchariflorus*. Cultivation of *M. x giganteus* is possible in areas where temperatures remain sufficiently high during winter, and high yields (18.7–36.8 t/ha) have been achieved [41]. Moreover, substantial yields (13–21 t/ha) were reported when cultivated on marginal soils [42,43,44]. The potential for phytoremediation of heavy metal-contaminated soils [45,46] and its ability to act as a carbon sink during its cultivational lifespan [25,47,48] clearly add to why *M. x giganteus* is considered as one of the most promising biomass crops. However, costly rhizome propagation [49], vulnerability to potential pests and diseases due to the absence of genetic variability [50,51,52,53] and lack of cold tolerance leading to severe losses in the first winter after establishment [41,54,55,56] are notable drawbacks of this specific accession.

Despite being a highly promising energy crop, a major drawback surrounding *M. x giganteus* biomass is the resistance of its cell wall against deconstruction, making it recalcitrant towards targeted conversion and valorization through biorefinery. The recalcitrance of the cell wall is directly related to the composition, structure and architecture of the molecules it contains. *M. sinensis* and *M. sacchariflorus* genotypes with lower recalcitrance, performing up to 50% better, have been identified [57]. 

A large number of applications have been described for miscanthus biomass, with the suitability for a given application ultimately being determined by the cell wall composition. Especially the composition of the secondary cell wall, consisting of cellulose, hemicellulose and lignin, is of importance, as it accounts for >90% of the dry matter of the plant biomass.

Some applications aim to use the whole biomass fraction, such as energy generation through combustion or fast pyrolysis [58,59] or the production of biomaterials such as composite polymers, concrete or fiber boards [60]. Other applications only target a specific fraction of the cell wall for conversion into high value products. Polysaccharide-driven biorefineries are the most well-known example in this context, striving to hydrolyze cell wall polysaccharides into constituent monosaccharides to be fermented to ethanol or methane [61,62] or converted to platform chemicals such as furfural or 5-hydroxymethylfurfural [63]. Alternatively, cellulose could also be used for manufacturing nanocrystals [64]. Although the (hemi)cellulosic parts are still mainly targeted in most lignocellulosic refinery processes, and lignin is therefore generally considered an inconvenient barrier against the conversion of the biomass polysaccharides, lignin valorization is expected to become increasingly important, with it being the most abundant natural resource of aromatic building blocks [65,66]. 

Ideally, each biomass component could be efficiently separated and isolated for further processing [67,68]. However, this requires pretreatment of the lignocellulosic biomass, since it is recalcitrant to this fractionation and degradation. The required pretreatment stringency remains the first and foremost bottleneck for the design of a green and economically feasible production chain [69,70], requiring both high biomass digestibility and pretreatment efficiency. A useful measure to this end is enzymatic saccharification, since it allows evaluation of the amount of released monosaccharides. 

Production of bioethanol and methane are among the best studied applications for lignocellulose feedstocks, as they were initially identified as the most promising value chains [71]. Their production makes use of different ways of enzymatic saccharification, either through the application of enzymatic cocktails containing endo- and exo-glucanases and β-glucosidases of fungal origin or through exposure of biomass to hydrolytic bacteria [72,73,74,75]. After the saccharification step monosaccharides generated for bioethanol production are fermented, followed by distillation of the produced ethanol [70]. Alternatively, for methane production the monosaccharides are converted into organic acids and alcohols by acidogenic bacteria, which are subsequently converted into acetate, that serves as a substrate for methanogenic bacteria to produce methane and carbon dioxide [72,73,74]. Genetic studies in the field of biomass digestibility or pretreatment optimization for miscanthus or other lignocellulose grasses often use one of these approaches as a way to assess the performance differences among diverse accessions or pretreatment conditions.

### 1.2. Improving Biomass Quality in Miscanthus and Breeding Efforts

Interest in breeding of miscanthus is relatively recent, especially when compared to other crops [30]. It is a time-consuming and laborious process as, due to the perennial nature of the crop, agronomically relevant traits, such as plant yield and biomass quality, can only be evaluated in a representative matter after a growth period of at least 2–3 years [76]. Within the genus *Miscanthus*, a large variability for the different traits contributing to cell wall quality is present, enabling selection and breeding for reduced cell wall recalcitrance based on knowledge of cell wall composition. However, cell wall quality is not easily assessed or captured during the breeding process as it is determined by many different traits that are polygenetic in nature.

Breeding starts with the availability, generation and search of genotypes with promising quality properties. In general, such genotypes do not have the best overall agronomic characteristics and need to be crossed with advanced breeding material to combine quality with other desirable characteristics, such as high-yielding potential. In practice, this implies a recurrent cyclic approach of crossings and selection to improve quality and agronomic performance and requires appropriate screening tools. The highly diverse germplasm available in *Miscanthus* are attractive sources for desirable cell-wall properties. For instance, natural *M. sinensis* populations from different geographical origins include six distinct genetic clusters and thereby the existence of potential heterotic groups that have so far remained unutilized [33]. Alternatively, deliberately created mutants obtained through targeted genetic modification or undirected mutagenesis could become an additional beneficial source of variation for cell wall genes. To discover useful quality characteristics from selected mutated plants, advanced breeding material and/or wild germplasm, they have to be clonally propagated, through plant splitting or tissue culture to establish replicated field trials for evaluation. The best performing genotypes could either be tested in multi-location trials for their potential as a clonally propagated cultivar, used as parental lines for production of hybrid seeds or serve as a source of beneficial genes for recurrent selection breeding [77,78].

The mating system of fertile species like *M. sinensis*, being gametophytic self-incompatibility (SI), influences the actual breeding in different ways. The system, most likely based on two multiallelic genes as is commonly found among grasses [79], prevents self-pollination but on the other hand it enables the use of heterosis. The breeding program at Wageningen University focusses on the latter and aims to breed for seed-based *M. sinensis* experimental hybrids through pair-wise crossings in isolation among selected genotypes. The SI system limits the full potential of hybrid breeding, but mating between either full sibs or half sibs can circumvent this limitation [80]. Emphasis of the Wageningen breeding program is on selection of candidate clones/individuals for making biparental crosses and on subsequent testing of full-sib families, in particular [81]. The ultimate goal is the creation of hybrid families suitable for commercial use. To remake the original families, the parental clones are maintained. Other breeding programs use similar approaches but instead aim mainly for interspecific seed-based hybrids [78]. Alternatively, creation of new clonally propagated “giganteus” varieties (*M. sinensis* x *M. sacchariflorus*) is also ongoing [82,83].

The use of molecular tools has been explored in miscanthus and resulted in the identification of genetic markers that could potentially speed up the breeding process dramatically. Mapping populations have successfully identified numerous QTLs contributing to important traits such as biomass yield and quality [84,85,86,87]. Additionally, genome-wide association studies in an experimental *M. sinensis* population showed the potential of genomic prediction and selection [88,89]. The use of sufficiently large populations to find SNPs corresponding to traits of interest requires phenotyping of a large number of plants. Analytical protocols for analysis of the main cell wall components and structural sugars have been commonly used for many years. Additionally, there are many more protocols available that make it possible to obtain detailed insight into the composition and structure of these components. While these methods provide information that could be critical for further advancing selection, they often require specialized equipment and are not considered as high throughput. Many of these traits have been successfully analyzed using infrared and near-infrared spectroscopy techniques, that are capable to predict the content of many cell wall structures and can be considered as high-throughput alternatives once appropriate calibration models have been created [90,91].

Identification of cell wall components that are favorable or detrimental for cell wall deconstruction and enzymatic hydrolysis has been the focus of different research programs in the last decade. In this review, we aim to provide an overview of the effects of different cell wall components on cell wall recalcitrance and the variation that can be found within different miscanthus species. Finally, we will discuss the many recommendations that have been made for breeding towards the improvement of biomass quality for bioenergy, polymers and chemicals and how these could be used within miscanthus breeding programs.

## 2. Cell Wall Composition in Relation to Cell Wall Digestibility

Miscanthus cell walls are highly complex structures that are largely comprised of lignocellulose with small amounts of pectin and proteins [92]. During cell expansion the primary cell wall, a thin relatively flexible layer that consists of cellulose, hemicellulose and pectin, is formed. Once expansion is completed, a secondary cell wall layer is deposited in sclerenchyma cells [93]. This secondary cell wall is a thicker structure in which cellulose and hemicellulose are bound by lignin, creating a reinforced hydrophobic network that provides additional strength and rigidity as well as protection against environmental and biotic stresses [94,95,96]. The cell wall is further strengthened by different crosslinks between the cell wall components.

Cellulose is the major component of mature *M. x giganteus* cell walls in stems (45–49% *w*/*w*), followed by hemicellulose (27–30% *w*/*w*) and (acid detergent) lignin (7–12% *w*/*w*) [97,98,99]. Differences in cell wall composition are largely determined genetically, although this could be influenced by environmental conditions and the plant’s developmental stage, as well as the analytical methods used [57,97,100]. The large genetic variation that is available within miscanthus is illustrated by a study that evaluated the cell wall composition of 510 accessions belonging to four different species, with a range of 26–54% (*w*/*w*) for cellulose, 18–43% (*w*/*w*) for hemicellulose and 5–19% (*w*/*w*) for lignin [101].

Screening accessions for biomass digestibility has resulted in the identification several *M. sacchariflorus* and *M. sinensis* genotypes with improved cellulose conversion rates of up to 50% compared to *M. x giganteus* [57]. Lignin is well-known to limit the saccharification process. Hence, its relatively high content in *M. x giganteus* compared to other accessions likely explains to a large extent the difference in conversion [61,102]. However, lignin content alone does not fully explain cell wall digestibility [103,104].

**Figure 1:**
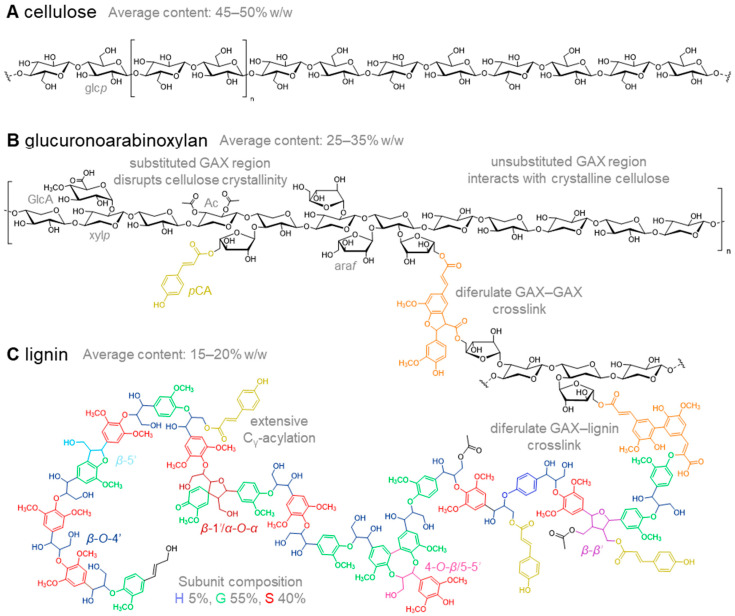
Schematic representation of the major constituents of the miscanthus secondary cell wall. The cellulose (**A**), glucuronoarabinoxylan (GAX) (**B**) and lignin (**C**) structures attempt to fairly represent the (relative) abundances of each individual structural moiety and the interactions between the polymers based on literature. Note that for simplicity only two types of diferulates (8-5 and 5-5) are shown, though many other linkage types are known to exist.

In this section, we will review in detail the different cell wall components (Figure 1) and discuss the effects of alterations in specific cell wall characteristics affecting biomass quality, here viewed as polysaccharide degradability. The relevance of the structural variation that can be found within the different species will be included, as well as the interaction and organization of cell wall components. Understanding the effects and complex interactions is essential for the identification of breeding targets for development of miscanthus varieties with improved biomass quality.

### 2.1. Cellulose in Miscanthus Cell Walls

Cellulose consists of a backbone of β-(1→4)-linked d-glucosyl moieties (Figure 1A) that are joined together in chains of up to several thousand and form the core structure that provides strength to the plant cell wall [105,106]. In general, cellulose makes up the largest proportion of the cell wall in miscanthus and values as high as 58.8% (*w*/*w*) have been reported [61,98,104,107]. However, in some genotypes with low cellulose levels (below 40% *w*/*w*) its content can be exceeded by hemicellulose [107].

In miscanthus, stems have a higher cellulose content compared to leaves, regardless of the species [61,108]. For instance, reported relative differences in cellulose levels between leaves and stems were 24.9%, 15.5% and 15.9% for *M. sinensis*, *M. sacchariflorus* and *M. x giganteus*, respectively [109]. Despite cellulose content being higher in stems, stems appeared more difficult to hydrolyze than leaves, as evidenced by lower glucose saccharification rates [110,111]. Therefore, cellulose content by itself cannot be considered as a predictor of saccharification efficiency. Different studies have reported low or no significant correlations between cellulose or glucose levels and enzymatic saccharification [61,111].

Enzymatic saccharification is affected by different characteristics of cellulose, including the degree of polymerization, crystallinity and the pretreatment it underwent [112]. Cellulose that was isolated from stem material had a higher degree of polymerization than that from leaves (800 vs. 580) [113], which might contribute to the lower observed saccharification rates in stem material. In addition, there seems to be an effect associated with stand age, as the degree of polymerization gradually increased over a five-year period from 880 to 1050 units [114]. Material that was harvested during the growing season had a higher degree of polymerization than when harvested after the growing season [115]. In unpretreated miscanthus stalks, the degree of polymerization varied between 957 and 1461, showing no correlation with cellulose release (maximum release ~15%). However, after pretreatment the degree of polymerization was found to be reduced in each accession (average reduction ~35%) and significantly correlated to hexose yield (maximum release ~40%) and was identified as one of the features that negatively affects enzymatic saccharification [116]. Another study established a negative effect of the degree of polymerization of the crystalline cellulose proportion of cell walls on hexose release yields [117]. 

A distinction can be made between structurally amorphous and crystalline regions, with the latter being far more resistant to degradation due to its highly ordered structure based on hydrogen bonds between cellulose polymers and van der Waals forces between glucose molecules [118,119,120]. Crystalline cellulose is hydrolyzed by exoglucanases in a processive, and therefore rather slow manner, while endoglucanases act on the more amorphous regions [118]. Therefore, reducing the level of crystallinity would facilitate enzymatic saccharification [121]. 

Large genotypic variation in the cellulose crystallinity levels (23.5–59.9% *w*/*w*) of raw miscanthus biomass from several species have been reported and could indeed be confirmed as a negative factor on the amount of hexoses released during enzymatic saccharification [117,122]. Studies on the cellulose fractions of other crops have reported similar findings concerning the negative effect of crystalline cellulose on enzymatic saccharification rates [123,124,125].

### 2.2. Hemicellulose Composition in Cell Walls of Miscanthus

Hemicelluloses are heterogeneous branched polymers consisting of multiple monosaccharides, that are essential in strengthening the plant structures to ensure normal growth [126]. Hemicellulose in miscanthus (~25–35% *w*/*w*) consists mainly of glucuronoarabinoxylans (GAX) (~30% *w*/*w*), a typical characteristic of grasses [61,127,128]. GAX is comprised of a β-(1→4)-linked xylopyranosyl backbone that is highly substituted with α-l-arabinofuranosyl (ara*f*) moieties (~3% *w*/*w*), mainly at α-(1→3)-, but also α-(1→2)-position (Figure 1B) [128]. The proportion of (4-*O*-methyl)glucuronyl units(GlcA), attached at the α-(1→2) position [92], is very low in miscanthus [107,129,130]. In addition, xylosyl moieties can be substituted by acetyl groups at the *O*-2 or *O*-3 position [131].

In grasses, *p*-coumaric acid and (di)ferulic acid can be esterified to the ara*f* units, with the latter being much more abundantly incorporated and playing an important role in intra- and inter-molecular crosslinking of the different cell wall structures [132,133]. In addition, there are several other minor components that complete the total hemicellulose proportion of the cell wall. For instance, minor amounts of galactose (0.9%) and mannose (0.5%) have been observed [134], while analysis of partially methylated alditol acetates (PMAAs) identified low proportions of 1,4,6-linked glycopyranose and 1,3-linked glycopyranose units that represent xyloglucan and mixed-linkage glucans or callose, respectively [128]. Relatively high amounts of callose (5%) and minor amounts of mixed linkage glucans (<0.5%) were reported in *M. x giganteus* leaves [135].

Hemicellulose composition was found to remain unchanged during the growing season until senescence occurred and can be considered as a constant factor irrespective of the plant growth stage [104]. There is no significant difference in xylose content between leaves and stems after senescence, while arabinose was higher in leaves (2.44% *w*/*w*) than in stems (1.32% *w*/*w*) [104]. Higher arabinose and similar xylose levels indicate that the degree of arabinose substitution is higher in leaves than in stems. For instance, in *M. x giganteus* the arabinose/xylose ratio in leaves (0.16) was more than two-fold higher than that reported in stems (0.07) [111]. 

A strong negative correlation (*R*^2^ = −0.94) between hemicellulose content and methane yields through anaerobic digestion has been reported [136]. Contrary to these results, no significant correlation was detected for hemicellulose and methane yields in another study [61], while a positive correlation between hemicellulose content and methane yields have also been reported in miscanthus [137]. Anaerobic digestion profiles of xylan showed that this polysaccharide was more difficult to hydrolyze and yielded a lower amount of methane than cellulose. However, simultaneous digestion of 1:1 mixtures of cellulose and xylose caused an increase in methane yields exceeding those of individual components by themselves [138].

In the absence of pretreatment, xylose content was negatively correlated with saccharification efficiency of glucose and xylose in miscanthus [111]. It is well known that xylan, xylose and their intermediate xylooligomers inhibit cellulase activity [139], which could explain this effect. On the other hand, when pretreatment is applied hemicellulose content is often considered as factor positively affecting enzymatic saccharification [61,110,122,140]. One explanation is that the hemicellulose portion is the most labile component and relatively easy to degrade by various pretreatments, leading to greater cell wall disintegration and higher exposure of cellulose-binding sites to cellulase. 

The positive effect of hemicellulose content has also been associated with a decrease in cellulose crystallinity [122,140]. More specifically, it was the amount of arabinose substitution that was negatively correlated to cellulose crystallinity and thus positively correlated to saccharification efficiency. In addition, when the xylan backbone contained a low amount of arabinose substitution there was a negative correlation to cell wall digestibility [140]. Li et al. [140] also detected xylose and arabinose after extracted residues were exposed to cellulases that are non-reactive for these polysaccharides, implying that there must be a close interaction with cellulose. Interactions between xylans and cellulose have often been proposed and were proven to occur via in situ analysis quite recently [141]. In vitro experiments have shown that a higher degree of arabinose and *O*-acetyl substitutions on the xylan backbone decrease the adsorption of xylans to cellulose [142,143]. However, such experiments also showed that the binding of arabinoxylan to cellulose has a very limited effect on overall cellulose macrostructure and crystallinity [144,145]. Simulation experiments indicated that binding is stabilized by substitutions at the α-(1→2) position, irrespective of the structure positioned there [146]. However, substitutions at the α-(1→2) position are low in miscanthus, as the amount of α-(1→3)-linked arabinosyl units is between 4 and 6 times higher compared to the amount positioned at the α-(1, →2) position in stems [128]. Results from another simulation study reported stronger binding of less substituted arabinoxylan to cellulose, but also showed strong binding of ferulic acid to cellulose, proposing that ferulic acid could play an important role in arabinoxylan to cellulose interactions [147]. Clearly, further research would be needed to confirm if such interactions occur in vivo in the cell walls of miscanthus.

### 2.3. Lignin in Miscanthus Cell Walls

Lignin is a structurally complex aromatic polymer, that is predominantly made up by three monolignols precursors that are synthesized through the phenylpropanoid pathway: *p*-coumaryl alcohol, sinapyl alcohol and coniferyl alcohol [148,149]. After incorporation into the lignin polymer, these moieties are referred to as *p*-hydroxyphenyl (H-unit), syringyl (S-unit) and guaiacyl (G-unit) units, respectively (Figure 1C). Polymerization of monolignols occurs through oxidative radical coupling under combinatorial control to form various aryl-ether and carbon–carbon interunit linkages. The β-*O*-4′ aryl ethers are by far the most abundant interunit linkage, generally accounting for 80% of all interunit linkages, with phenylcoumarans (β-5′), resinol (β-β′), dibenzodioxocins (4-*O*-β/5-5′) and spirodienones (β-1′/α-*O*-α) completing the major linkage motifs [148,150,151]. Besides these three monolignols, hydroxycinnamic acids and acetate are incorporated into grass lignins. In grasses, *p*-coumaric acid, acetate and traces of ferulic acid, can be acylated to the monolignols at the C_γ_-OH position prior to incorporation into the lignin polymer [93]. As a consequence of the “blocking” of the C_γ_-OH position, resinol substructures cannot be formed anymore, and instead tetrahydrofuran structures are formed in lignins with high acylation extents [152]. Ferulic acid is majorly incorporated through the same radical coupling mechanisms occurring between monolignols and is the major origin of lignin–carbohydrate complex formation in grasses [153]. Next to hydroxycinnamates, the flavonoid tricin is incorporated into the lignin of grass biomass sources, including miscanthus, albeit at relatively low levels [154]. Interestingly, the lignin of C4 grasses differs substantially in structure from that of C3 grasses, with the former generally being much richer in *p*-coumaric acid moieties [152,155,156].

Considerable variation in lignin content can be found within and between stems of different miscanthus species, varying from less than 5% (*w*/*w*) to just over 18% (*w*/*w*); again, in absolute terms, this is heavily dependent on the method used for determination [101]. *Miscanthus sinensis* accessions have on average lower lignin levels compared to those belonging to *M. sacchariflorus* or *M. x giganteus* [57,101]. It is well established that lignin levels increase during the growth season [61,100,157]. For instance, summer-harvested miscanthus genotypes had on average 34% lower relative lignin levels compared to those harvested in early spring [61]. In addition, histological staining of internodes in *M. lutarioriparius* also visualized the maturation effect as the second internode was highly lignified, while in the younger 11th internode lignification was largely absent [157]. Furthermore, stems are usually slightly more lignified than leaves. After senescence, the lignin levels in stems were found to be on average 1.15% *w*/*w* and 1.70% *w*/*w* higher than in leaves [100,104]. 

Lignin contributes to cell wall recalcitrance by acting as a physical barrier preventing the accessibility of hydrolytic enzymes to polysaccharides. In addition, lignin is able to irreversibly adsorb these enzymes; in this way, the hydrolysis of cell wall polysaccharides is also limited [158,159]. Results from a recent study indicate that the effect as a physical barrier is of higher importance in grasses, as even though adsorption occurred, it appeared not to limit the enzymatic activity [160]. In either case, it is well established that lignin is the foremost limiting factor for effective cell wall deconstruction. A study that evaluated 41 plants belonging to 11 different crops reported that lignin content could explain 80% of sample variation for biogas yield [161] and it is of no surprise that lignin content is also the main limiting factor for the conversion of biomass into ethanol or methane in miscanthus [61,102]. Lower lignin content in summer-harvested material and leaves are likely to contribute to more efficient degradation, improved saccharification rates and higher biogas yield compared to winter-harvested miscanthus and stems [110,111,137].

In depth structural analyses have revealed that lignin in miscanthus consists largely of G-units and S-units with a minor number of H-units, as is typical for grasses [92,128,130,162]. For example, Hage et al. [162] reported the monolignol molar ratios for *M. x giganteus* to be 52% G-units, 44% S-units and 4% H-units. A parameter often used to evaluate lignin composition is the S/G ratio, as H-units compose only a small part of the total lignin polymer. Between studies, the S/G ratio of *M. x giganteus* ranged from 0.54 to 0.84 [128,162,163,164]. A similar range in S/G ratio (0.33–0.70) can be found between highly different species, as for instance was reported by Schäfer and co-workers [128]. Interestingly, the lignin composition also differs greatly between stems and leaves of miscanthus [100,108,128,165]. Lignin in leaves contains considerably more G-units than the lignin in stems. For instance, in *M. x giganteus*, leaves have a S/G ratio of 0.09 and for stems this was 0.54 [128]. Higher contents of G-units in leaves compared to stems have also been reported in other grass species, such as *Arundo donax* and wheat [166,167]. The most abundant interunit linkage encountered in miscanthus lignins is the β-*O*-4′ aryl ether, with ranges for *M. x giganteus* having been reported between 78 and 93% of the relative abundance. The remainder of the linkages consist of, in descending order: phenylcoumaran, resinol, spirodienone and dibenzodioxocin [128,163,164]. Variation on the amount of β-*O*-4′ linkages has been reported, as *M. sinensis* cultivar “Goliath” had 59.6% of such linkages, while *M. x giganteus* showed 77.6% relative abundance of the β-*O*-4′ motif [128]. The higher abundance observed for the latter miscanthus line presumably directly relates to a higher incorporation of S-units, which do not allow the formation of condensed moieties [168]. However, it must be noted the study by Schäfer et al. [128] showed no clear correlation between S/G and β-*O*-4′ abundance.

While the effect of total lignin content is clear, the contribution of lignin structure to cell wall recalcitrance is not as clearly established [158]. Even though it has long been believed that S-units are more susceptible to degradation because their dimethoxylated aromatic ring prevents the formation of strong “condensed” β-5′ and 5-5′ substructures, this notion is still under strong debate [169]. Indeed, contradicting observations are constantly being added to the pool of available literature on this topic.

Monolignol ratios can be altered by mutations of key components within the lignin biosynthetic pathway. As such, Li et al. [170] used genetically modified plants of *A. thaliana* with lignin mainly composed by either G-units (95.2%) or S-units (90.7%) and compared them with the wild type (G-units 76.2%, S-units 17.8%). Their results show enhanced digestibility within the mutant line containing mostly S-units, while the mutant with enhanced G-units had increased recalcitrance compared to both the other mutant line and the wild type [170]. A study on the influence of differences of lignin structures in the inner and outer tissues of *Erianthus* and sugarcane stems on cell wall deconstruction also showed that walls with lignin rich in S-units are more easily deconstructed than those rich in G-units [171].

It should be noted that results obtained in dicots are not necessarily meaningful for grasses because of their very distinct lignin structures, especially regarding the incorporation of moieties other than the canonical monolignols, and that even the comparison of studies between different grasses are difficult to compare due to the wide range of variation in experimental conditions and methods used [172].

For miscanthus the number of studies that investigated the effects of monolignol ratios on cell wall digestibility is somewhat limited. However, two studies reported that a higher proportion of S-units compared to G-units (increased S/G ratio) negatively affected saccharification efficiency and thus ethanol yields [173,174]. It was observed that biomass with a high proportion of G-units could be completely hydrolyzed, which was not the case for samples that contained a high proportion of S- or H-units [174]. Similarly, in maize an increased S/G ratio in stem lignin was shown to correlate to a decrease in rumen fermentation. The correlation (*R*^2^ = 0.80) was even higher than that with lignin content as such [175]. This study suggests that the composition of lignin is more important for cell wall deconstruction than merely its content. If this is true, the difference in digestibility usually found between stem and leave materials is likely in part caused by a difference in lignin composition [100,104,108]. Transgenic lines of several grasses (maize, switchgrass, ryegrass, sugarcane) with downregulated caffeic acid 3-*O*-methyltransferase (COMT) showed enhanced saccharification efficiency and ethanol production [176,177,178,179,180]. In these lines, not only is the S/G ratio lower but the total lignin content is also lower, and therefore the observations cannot be unambiguously pinpointed. 

### 2.4. Content and Structure of Pectin in Miscanthus Primary Cell Walls

Pectin is a class of complex polysaccharides that occur almost exclusively within the primary plant cell walls and middle lamella and are thought to be involved in essential processes as plant growth and development, cell expansion and cell wall structure and porosity [181,182]. Pectins are diverse polysaccharides falling into three structure types, homogalacturonan (HG) and rhamnogalacturonan I (RG-I) and II (RG-II) [183]. The most abundant pectins in plant cell walls are HG (around 65% *w*/*w*), a linear polymer of α-(1→4)- linked galacturonyl units that is partially methyl-esterified and acetylated [181]. RG-I, responsible for 20–35% (*w*/*w*) of total pectin content, consists of recurring structures of α-(1→4)-d-galacturonyl– (1→2)-l-rhamnosyl blocks, in which the rhamnosyl units can be substituted with (branched) α-(1→5)-arabinan units or β-(1→4)-galactan side-chains [181,184]. The most complex pectin structures belong to RG-II, consisting of a galacturonic acid backbone that has five types of complex side chains consisting of 12 different types of sugars and a variety of linkages [181,184].

Miscanthus cell walls only contain minor amounts of pectin, as is commonly the case in grasses. Additionally, in miscanthus HG and RG-I were identified as the main pectin structures [103]. Total uronic acid contents, including both galacturonic and glucuronic acid, have been reported to be 1.2% (*w*/*w*) of raw biomass and 1.8% (*w*/*w*) of total polysaccharide content [185,186]. Reported rhamnose levels in miscanthus have usually been very low (<0.30% *w*/*w*) [122,134,140] or even undetectable [187,188]. In other studies, total pectin levels (0.5–3.5% *w*/*w*) have been expressed as percentage of cell wall extracted with ammonium oxalate [140,187]. However, the levels found in this way probably overestimate the actual pectin levels, since ammonium oxalate extraction releases relatively large amounts of glucose and xylose compared to the typical pectin composing monosaccharides [103,129]. 

Although pectin content in miscanthus is usually very low, several studies indicate a positive relation of pectin levels with saccharification efficiency. A positive contribution of pectin content to saccharification efficiency after alkaline or acid pretreatment was attributed to the content of uronic acids. Higher levels of uronic acids were found to be associated with a decrease in lignocellulose crystallinity (*R*^2^ = 0.34) [129]. Removal of the uronic acids by ammonium oxalate extraction increased cellulose crystallinity between 1% and 6%, with samples higher in extractable uronic acids showing a larger increase in cellulose crystallinity (*R*^2^ = 0.58) [129]. Furthermore, growing of miscanthus on soil with cadmium was found to result in biomass with lowered levels of crystallinity, that the authors partly attributed to an increase in pectin content [187]. De Souza and co-workers [103] reported that specific pectin epitopes related to RG-I and arabinogalactan can have a positive effect on saccharification efficiency, while different epitopes belonging to the same structures contributed to recalcitrance. The authors suggested that the different pectin epitopes are most likely closely associated to different cell wall structures, as such it could be expected that epitopes contributing to recalcitrance are most likely closely associated to lignin [103]. 

In switchgrass plants, downregulated galacturonosyltransferase 4 (GAUT4) expression caused a decrease in HG and RG- II content and showed improved saccharification efficiency, caused by increased porosity of the cell wall due to a lack of crosslinking between these two pectin polymers [189]. Reduced GAUT4 expression also affected other cell wall parameters that likely favored saccharification, such as increased arabinose and xylose content, lower levels of hydroxycinnamic acids and reduced crosslinking between lignin and arabinoxylan [190]. More studies are supporting the hypothesis that pectin could be involved in crosslinking lignin to hemicellulose, the existence of such interactions between pectin and other cell wall components would explain why suppression of GAUT4 has such a large impact on the cell wall composition and digestibility in switchgrass [190].

### 2.5. Crosslinking of Polymers in Miscanthus Secondary Cell Walls

Crosslinking between different cell wall polymers enhances the overall strength of the cell wall matrix. Several types of covalent and noncovalent interactions occur within but also between the different cell wall components, for instance cellulose–cellulose, hemicellulose–hemicellulose and cellulose–hemicellulose interactions have been reported [191]. In grasses, dimerization of ester-linked hydroxycinnamic acids (ferulate) makes it possible to crosslink arabinoxylan polymers to each other photochemically and also through oxidative radical coupling [192,193]. Additionally, the ability to undergo radical coupling reactions causes ferulic acid or diferulic acid to become incorporated into the lignin polymer, facilitating crosslinking between arabinoxylan and lignin polymers [194,195]. Such (di)ferulic acid crosslinks are thought to play an important role in cell wall stiffening, regulation of cell expansion and to provide a barrier against pathogens and insects [194]. Furthermore, it has been shown that these crosslinks have a negative impact on cell wall degradation [194]. Reducing GAX–ferulate–lignin crosslinking improved fermentation in artificially lignified cell walls [196]. These linkages are rather labile and easily broken down during (alkaline) pretreatments [153,194]. As such, for pretreated *M. sinensis* biomass, positive correlations were reported between the extent of feruloylation and the amount of monosaccharides that could be released from the cell wall polysaccharides [61].

## 3. Interdependence of Biomass Quality and Pretreatment Efficiency

Pretreatment of lignocellulose biomass is an essential step in disrupting the native plant cell wall structure and enable the access to polysaccharides by hydrolytic enzymes, and to separate lignin and (hemi)cellulose fractions to make them available for further processing. Miscanthus biomass reacts poorly to enzymatic saccharification without a preprocessing step. Conversion efficiencies of cellulose to glucose from raw biomass have been reported to lie within a range of less than 3% up to 10% for *M. x giganteus* feedstock, which could be enhanced to 30% up to 80% depending on the type of pretreatment [197,198,199].

The available pretreatment methods rely on physical, thermal, chemical or biological techniques, and each allow for a distinct disruption of the plant cell wall [200]. Currently, such pretreatments are still a bottleneck for cost-efficient and carbon-neutral production of bioethanol and biopolymers [201]. For instance, evaluation of five different pretreatment methods in *M. floridulus* enhanced methane yields upon anaerobic digestion in the range of 10.2 to 41.1%. The highest improvement of 41.1%, due to NaOH pretreatment, may seem impressive, but did not result in lower methane production costs; actually they were 31% higher (0.448 USD/m^3^ vs. 0.587 USD/m^3^) [202]. The higher price/m^3^ was associated with costs for the chemicals and energy used for heating. This may also imply that feedstocks with biomass composition optimized for specific types of end uses require less stringent and costly pretreatment conditions for effective enzymatic hydrolysis [203]. As such, understanding how different pretreatments affect cell wall deconstruction could be translated into new criteria for selection to be used in dedicated breeding programs for the development of varieties with less recalcitrant biomass. Additionally, the prospect of reducing pretreatment intensity would result in a less severely modified lignin component, potentially opening up new valorization routes. In this section, we will discuss the effects of pretreatments on the biomass composition of miscanthus.

### 3.1. Hydrothermal Pretreatment 

Hydrothermal pretreatments are usually performed by bringing water in the absence of any added catalyst to temperatures between 160 and 240 °C, and therefore require the application of pressure to keep water in its liquid, rather than steam or gaseous state [204]. Cell wall degradation only occurs above a certain threshold temperature, and increasing the temperature is usually more effective than extending the duration of the pretreatment [205]. Prolonged exposure (10 h) of *M. floridulus* biomass to a moderate temperature of 95 °C showed degradation of hemicellulose and cellulose by only 5.0% and 1.3%, respectively. Degradation of lignin was not observed under these conditions [202]. Temperatures of at least 175 °C were needed to degrade a signification portion of hemicellulose, while the amount of degradation was further increased when temperatures were elevated to 200 °C [206]. Li et al. [207] gradually increased pretreatment temperatures, which was followed by rapid cooling after a certain threshold was met. This pretreatment caused hemicellulose degradation only at a temperature above 200 °C; at 230 °C, the removal of the fraction was complete in *M. lutarioriparious* [207]. The arabinose substitutions were more effectively degraded than the xylan backbone itself, while acetate, mixed-linkage glucans and glucuronic acid were also effectively hydrolyzed during hydrothermal pretreatment [208,209]. In fact, the released acetate could be considered an endogenous catalyst, of which the effects on hydrolysis should not be neglected in biomasses like miscanthus that can contain substantial levels of this acid, initially esterified to the cell wall constituents. As such, even though catalysts might not be added during “pure” hydrothermal pretreatments, they should not be considered catalyst free [210]. Other components, such as pectin and arabinogalactan were easily extracted at even mild conditions and were therefore identified as the most easily digestible fractions within the cell wall, in a study done on poplar [211]. As temperatures of 200 °C and above can totally dissolve hemicellulose it is not surprising that ester- and ether-linked hydroxycinnamic acids, that connect hemicellulose and lignin, were also largely dissolved [206,212].

Detectable alterations of the lignin polymer start at temperatures from 160 °C, while some isolated lignin fractions required temperatures from 180 °C for the disruption of interunit linkages to be initiated [212]. Hot water (160 °C) flow through treatment (25 mL/min) of *M. x giganteus* was found to cause a decrease in the S/G ratio of the remaining lignin, showing that syringyl units were preferentially removed in comparison to their guaiacyl analogs, which could be the result of the fact that a relatively higher fraction of said S-units is incorporated into the more labile β-*O*-4′ bonds [213]. Chen et al. [212] observed similar results, and found preferential degradation of S-units compared to other lignin units at 200 °C. In wheat straw pretreatment temperatures above 190 °C digested β-*O*-4′ bonds almost completely, while β-β′ units could still be detected and the β-5′ linkages remained unaffected [214].

Besides breaking of linkages, high temperature treatments also partially dissolve lignin, leading to the formation of lignin droplets that relocate and have the ability to deposit on various parts of the cell wall [215]. Li et al. [216] were able to confirm that the lignin droplets consisted out of a higher amount of S-units than G-units. Formation of such droplets could block accessibility of enzymes to other cell wall structures such as cellulose, but the effect of cell wall disintegration is expected to exceed the overall effect of potential blocking and as such would result in a net increase in cellulase accessibility [215]. In miscanthus, the formation of such lignin droplets has also been observed during dilute acid pretreatment at a temperature of 170 °C which was proposed to have similar effects [198,217]. In vitro experiments established that the occurrence of deposition of lignin droplets on the cellulose surface somewhat impaired enzymatic hydrolysis by forming a physical barrier, and that these effects exceed that of the adsorption of cellulase to lignin droplets [216].

Cellulose remains largely unaffected by hydrothermal pretreatment. However, weakly bound cellulose oligomers can still be released from amorphous cellulose at temperatures of 100 °C and above, while breaking of glyosidic bonds started at 150 °C. Crystalline cellulose only starts degrading at 180 °C due to its tight and compact structure [218]. Pretreatment of pure cellulose at 200 °C for one hour released 10% of the total cellulose content [218]. This is in accordance with results from Li et al. [207], who recovered approximately 92% of total cellulose content after severe pretreatment of miscanthus biomass. Logically, dissolving of the amorphous cellulose and the hemicellulose proportion increases the overall crystallinity levels of the remaining sample. However, this increase has little effect on total saccharification efficiency or biogas production [206].

### 3.2. Dilute Acid Pretreatment

Acid pretreatment mainly solubilizes the hemicellulose fraction by acid hydrolysis of glycosidic linkages and makes cellulose more accessible to enzymes [217]. Dilute acid pre-treatment is usually the preferred method as the use of concentrated acids is (product-wise) associated with increased formation of carbohydrate-derived byproducts (e.g., furfural and hydromethylfurfural) and process-wise it is more expensive and requires more frequent maintenance of equipment due to corrosion [204]. Increased temperatures are required for effective disruption of the cell wall structures when dilute acids are used [219,220]. 

Miscanthus pretreated with 1.1% H_2_SO_4_ at a temperature of 122 °C was shown to solubilize 71% of the total xylose content, while retaining 73% of the original solid content containing 94% of the initial glucose content [221]. Higher acid concentrations (4% H_2_SO_4_) extracted between 77% and 87% of hemicellulose [222]. At different pretreatment severities, arabinose decreased at a higher rate from the hemicellulose fraction than xylose [198]. In a study with corn stover, it was also observed that arabinose needs less severe conditions to become totally dissolved than xylose. Release of the latter increased with pretreatment severity, therefore, it was concluded that arabinose linkages were the most labile during acid pretreatment [223]. However, similar hydrolysis rates for arabinose and xylose have also been reported in miscanthus [197]. 

Reductions in *p*-coumaric and ferulic acid upon pretreatment of miscanthus were 49.2% and 29.8%, respectively [198]. On the other hand, though ferulate levels were extensively reduced in rice straw, *p*-coumarate levels remained mostly unaffected [224]. Not only hydrolysis and release of the hydroxycinnamic occurred, but lignin removal was estimated to be between 5 and 13% (*w*/*w*) depending on pretreatment severity [198], while another study showed a maximum removal of almost 30% [222]. Moxley et al. [223] observed a larger decrease in S-units within lignin with increased pretreatment severities than of G-units, which could indicate that S-units were more easily hydrolyzed by the dilute acid pretreatment, though a preferential condensation of G-units cannot be excluded under the used conditions. Preferential hydrolysis of S-units has also been implied in switchgrass, which coincided with more extensive degradation of β-*O*-4′-linkages compared to their β-β′ and β-5′ analogs [225]. A heavy depletion of β-*O*-4′-linkages as a consequence of dilute acid pretreatment has been observed in various other lignocellulosic feedstocks, such as rice and wheat straw [214,224]. 

### 3.3. Alkaline Pretreatment

Alkaline pretreatment results in a partial deconstruction and solubilization of lignin from the cell wall, mainly by cleaving ester bonds between hemicellulose and lignin, as well as ester and ether bonds within lignin itself [226]. Advantages of this type of pretreatment are the potential to be effective at moderate conditions regarding temperature and pressure required contrary to other methods such as hydrothermal or acid pretreatment [226]. 

Lignin deconstruction is considered to be the main mechanism of cell wall disruption during alkaline pretreatments and resulted in a removal between 51% and 72% upon pretreatment with 4% (*w*/*v*) NaOH at 50 °C with a 2 h pretreatment time [222]. Pretreatments differing in severity regarding NaOH concentrations (0.5, 1.0 and 1.5% *w*/*v*) and time (15, 30 and 60 min) resulted in extraction between 42% and 85% of lignin [227]. Jung et al. [227] observed an accompanying decline in the H/G ratio from 0.48 in raw material to 0.13 in pretreated biomass and therefore suggested preferential degradation of H-units compared to G-units. Care must, however, be taken in the interpretation of these outcomes, because the methods used do not allow distinguishing “true” H-units from *p*-coumaric acid, of which the latter is logically very labile at alkaline conditions being entirely present as C_γ_-esters. Complete degradation of H-units (~4% of the total lignin polymer) was, however, also shown when using NH_4_OH pretreatment and determined by HSQC NMR analysis, able to differentiate H-units and *p*-coumarates [228]. S-units seem to be slightly less degradable than G-units when pretreating miscanthus biomass using alkaline chemicals, as their reduction (40%) was lower than that of G-units (53%) [228]. This finding is in accordance with the slight increase for the S/G ratio from 0.64 to 0.77 as was observed by Jung et al. [227]. Reduction in linkages between lignin units was ~50% for β-*O*-4′, β-β′ and β-5′, and 85% for the /4-*O*-β/5-5′ linkage [228], which is likely due to the phenolic nature of the latter dibenzodioxin linkage. The presence of such free phenolic groups likely lies in the basis of lignin solubility in alkali [229,230]. Hence, it can be conceived that lignins, or lignin populations, richer in phenolic/terminal units, will be more susceptible to removal by alkali. As an effect of the interunit linkages that (can) form between the different subunits, H- and G-units overall occur relatively more as phenolic units in comparison to their S-unit analogs [150,231]. However, these non-etherified linkages are condensed (5-5′ or 4-*O*-5′) and, as a consequence, are more resistant against cleavage or conversion [150]. Since multiple structural effects are at play at the same time, the overall susceptibility of lignins differing in subunit composition towards alkali treatment remains, hitherto, difficult to predict [150].

The polysaccharide—lignin crosslinks mediated by ferulic acid content are completely dissolved during alkaline pretreatment [171,174,228]. The accompanying degradation of hemicellulose itself varied between 4.6% at the most mild conditions (0.5% NaOH, for 15 min) and 32.5% at maximum tested severity (1.5% NaOH, 60 min) [227]. Cellulose degradation hardly takes place, even under more severe pretreatment conditions [227,228].

### 3.4. Pretreatment Efficiency

In the previous sections, we discussed three pretreatment methods that are commonly used in studies focused on cell wall digestibility. In recent years, however, many other methods and combinations thereof have been evaluated in miscanthus to optimize biodigestibility (Table 1). 

Despite all of this work, it remains a challenge to choose the most appropriate pretreatment for miscanthus biomass. Kumar and Sharma [239] highlighted that the selection of an appropriate pretreatment should be based on the composition of the biomass, as there is no one-size-fits-all solution available. Although we only focused on cell wall deconstruction here, over-degradation of polysaccharides and lignin can lead to the formation of fermentation inhibitory substances, such as weak acids, furfural, hydroxymethylfurfural and phenolic compounds [240]. An optimized pretreatment should ensure the highest possible level of saccharification and therefore find a balance between high levels of cell wall degradation while avoiding over-degradation [241,242].

Only a few studies are available that have examined the effect of more than one pretreatment on a range of miscanthus genotypes. Several studies have reported that sugar release after alkaline pretreatment was higher than what could be achieved with dilute acid pretreatment [117,122,243]. Another study reported minor differences in terms of output between these two pretreatment methods, with some genotypes releasing slightly more glucose upon dilute acid treatment and others upon alkaline pretreatment, hence suggesting key differences in their cell wall composition and/or architectures [97].

The cell wall composition determines the amount sugars released upon pretreatment. Glucose release of *M. sinensis* and *M. sacchariflorus* genotypes has been between 32.5 and 50.0% higher compared to *M. x giganteus* after dilute acid-, alkaline- and steam-explosion pretreatment [57,107,244]. Higher saccharification rates under equal pretreatment conditions result in a higher production of for instance ethanol, but also indicate less severe pretreatments conditions could be used [203]. Reducing the pretreatment intensity can be seen as a clear advantage since most of the pretreatments’ costs are related to the energy and chemicals that are needed [245]. However, in practice, potential biofuel yields of a feedstock depend on both biomass yield and saccharification efficiency. Results from a field trial in Poland showed higher ethanol yields from an *M. sinensis* variety compared to *M. x giganteus*. However, the potential of total ethanol production was slightly higher for *M. x giganteus* as it produced more biomass [246]. Obviously, this means that accessions that would combine the improved cell wall digestibility of *M. sinensis* with the yields of *M. x giganteus* would be superior and reduce the costs of biofuel production. 

## 4. Breeding for Improved Feedstock Quality

In recent years, research programs that focused on biomass quality in miscanthus have provided a number of traits that would improve the quality for different applications. The outcome of such work provided a good basis for future breeding programs, a matter that will be discussed in depth in the following section. Nonetheless, we will only focus on those traits that directly affect cell wall digestibility, and will not discuss aspects, such as harvesting regimes or agricultural practices, that could be used to obtain a more digestible feedstock.

### 4.1. Breeding for More Digestible Cell Walls 

Improving digestibility of plant material means that it is necessary to obtain genotypes containing relatively lower proportions of recalcitrant structures and higher proportions of labile structures either through breeding or biotechnology. Since lignin has been identified as the key factor limiting digestibility, lowering its content is often pointed out as a key breeding target [61,107,137]. Lignin content is a genetically complex trait, as illustrated by the 14 major genes that were found to be involved in the lignin biosynthetic pathway in *M. x giganteus* [247]. In their study, Zeng et al. [247] proposed a biosynthetic model of the genes belonging to the general phenylpropanoid- and monolignol-specific pathways. Such a high amount of genes involved is likely to coincide with the large number of QTLs that were detected for acid detergent (6) and Klason lignin (11) content in a biparental *M. sinensis* mapping population [86]. Several studies have reported high heritability values for lignin content in miscanthus [86,88]. These results, together with the large genetic variation that is available in miscanthus, make breeding for lower lignin content achievable through both conventional and marker-assisted breeding.

It is suggested that a higher proportion of specific lignin structures contribute to saccharification and are therefore interesting breeding targets. This potential was further illustrated by research performed on a population of recombinant inbred rice lines, in which a large number of significant QTLs (22) related to lignin composition or sugar release were detected. Four of these QTLs were corresponding to both lignin monomer composition and biomass digestibility. Lines containing all four of these QTLs had similar lignin content but with a lower proportion of S- and G-units compared to the other lines, and showed an increase of 19.3% and 36.4% for glucose and xylose release, respectively [248]. Screening of wild germplasm showed variability for monolignol ratios in miscanthus [173], indicating that genetic differences are likely to occur and could be utilized in breeding programs. Although an exceptional amount of either lignin monomer might enhance the digestibility under specific pretreatment conditions, and could thus be targeted, the conflicting results that have been reported make it difficult to formulate strong recommendations for breeding towards S-unit- or G-unit-enriched lignin content at this point. 

Alternatively, manipulation of different genes within the lignin biosynthetic pathway has shown to be a promising approach, and has resulted in the creation of lower lignin level mutants for a number of grasses [172]. Generally speaking, downregulation of phenylalanine ammonia lyase (PAL), cinnamic acid 4-hydroxylase (C4H), hydroxycinnamoyl-CoA:shikimate hydroxycinnamoyl transferase (HCT) and *p*-coumaroylshikimate 3′-hydroxylase (C3′H) induces a decrease in lignin contents, while downregulation of ferulic acid 5-hydroxylase (F5H) and caffeic acid *3*-*O*-methyltransferase (COMT) lowers the proportion of S-units within the mutants [249]. In the past, many of such mutants displayed impaired growth, either due to a lower ability to withstand the pressure during transpiration or interference with multiple processes that are depending on specific genes in the phenylpropanoid pathway that are essential for normal plant growth [249]. As such, downregulation of COMT gene did indeed lead to smaller plants with decreased lignin levels in *M. sinensis*. The highest observed absolute reduction in lignin for leaves and stems were 23% and 9%, respectively [250]. In contrast, downregulation of COMT in switchgrass did not affect plant biomass, while lignin content not only decreased but its composition was also altered as the S/G ratio changed from 0.69 in control to 0.37 and 0.39 in mutated lines [176]. These changes in lignin quantity and composition caused an increase in saccharification efficiency of whole plants between 16.5% and 21.5% after mild pretreatment, while ethanol conversion of stem material increased by 25% compared to the control plant. In both of the studies by Yoo et al. [250] and Fu et al. [176], the polysaccharide content of transgenic lines was either moderately affected or not affected at all. Moreover, a two year field trial confirmed that the COMT mutated switchgrass lines also performed better after senescence, as sugar release in the second year increased by 32.0% and 34.2% [177]. Other genes (4CL, CCR, CAD, PMT) have also been successfully altered leading to lower lignin content without hampering biomass yield [172]. However, so far genetic transformation in miscanthus has been challenging and these genes have not been manipulated in miscanthus, therefore further development is needed in this area. 

Inducing random mutations using radiation is an alternative approach for creating variation that circumvents the need of advanced transformation techniques [251]. The downside of such an approach is the large number of mutated plants that need to be screened, as most mutations will result in no or undesired effects. Wang et al. [251] used heavy-ion irradiation and were able to identify a promising mutant with lower lignin content and higher saccharification efficiency without a reduction in yield. Although the genes that were altered could not be identified, they mention it was likely, given the red stem phenotype that several lignin, flavonoid and anthocyanin pathways were affected.

To keep up with the advancements in the field of breeding and genetic modification, the analytical toolkit for evaluating lignin content and detailed structure needs to likewise advance. Hence, recent developments that allow the analysis of the whole cell wall in the gel state by HSQC NMR and quantitate lignin in submilligram sample sizes by py-GC-MS, now call for employment in further assessing digestibility in relation to the lignin present [252,253].

### 4.2. Improving the Polysaccharide Composition to Enhance Cell Wall Digestibility

Hemicellulose has been considered as a positive factor that contributes to easier cell wall deconstruction when pretreatment is applied, therefore selecting genotypes with a high hemicellulose content has been proposed as another strategy to increase the digestibility of miscanthus biomass [61,127,137]. On the other hand, as hemicellulose removal during pretreatment greatly enhances the saccharification efficiency, it can also be considered as a factor that causes recalcitrance [254]. For instance, xylose content correlated negatively to polysaccharide when no pretreatment was applied [104]. A more in depth evaluation of the hemicellulose portion revealed that arabinose substitution of the xylose backbone seems to be especially beneficial and was proposed as a prospective target for genetic manipulation [140]. Two rice mutant lines with increased expression of two glycosylrtransferase 61 (GT61) genes (XAT2 and XAT3), involved in xylan arabinosylation, showed higher levels of arabinose substitution concomitantly displaying a decrease in cellulose crystallinity and improved saccharification rates [255]. Genetic variation for total hemicellulose, xylose and arabinose content has been observed in a number of studies [127], while also a large number of QTLs have been detected for these traits in *M. sinensis* [86]. Manipulation of the GT61 gene XAX1 altered the arabinose substitution pattern on xylose, however, it also largely reduced the amount of ferulic acid. It was proposed that the reduction in ferulic acid content would largely explain the increased saccharification content [256,257]. On the other hand, ferulic acid has also been mentioned as a potential breeding target for enhancing biomass degradability during alkaline pretreatments [61,258]. Suppressing the expression of GAUT12 induced a decrease in xylose and pectin levels causing an increase in saccharification efficiency [259]. Pectin polysaccharides represent only a little amount of the cell wall, but seem to contribute to cell wall digestibility in one way or another [103,129,189]. Up to now, the exact role of pectin on saccharification efficiency remains unknown.

Since miscanthus has hardly been domesticated, the genetic variation that is available in nature can be seen as the most important source for future breeding. Recurrent selection programs that would select for lower lignin content and a high proportion of highly substituted arabinoxylan would gradually result in advanced breeding material with decreased cell wall recalcitrance. Given the time frame needed for reliable trait assessment the development of reliable genetic markers for the main traits affecting biomass quality are important and could speed up the breeding progress dramatically. In addition, the development of more advanced high throughput methods, for instance by establishing NIRS calibration models for complex traits, remain important to aid further breeding.

## 5. Conclusions and Future Perspectives

Efficient deconstruction of the plant cell wall remains an economic hurdle for applications that rely on the utilization of specific cell wall components. Miscanthus cell walls exhibit complex interactions between the different cell wall components and while a lot of knowledge has been gathered, their effect on cell wall digestibly is not yet fully understood. It is clear that accessions that possess lower lignin contents respond better to enzymatic hydrolysis of the polysaccharide fractions. Since the breeding interest in miscanthus has been relatively recent, and at the moment the alternatives to *M. x giganteus* are still limited, a significant improvement could be expected in the short term from utilizing the natural genetic variation available within the species for the selection of high yielding varieties with lower lignin contents. Targeted breeding and genetic modification approaches will without a doubt further contribute to the creation of less recalcitrant biomass in the long term. However, pretreatment steps are likely to remain critical and advancements in this area will further aid the efficiency of the total lignocellulose valorization chain. Therefore, increasing the content of the more labile fractions in the cell walls would be sensible breeding targets. For miscanthus, the results from the literature suggest that this would mean breeding for a highly arabinose-substituted xylan backbone and further increasing the content of minor polysaccharides within the cell wall would also have positive effects. Reducing lignin–polysaccharide crosslinking by decreasing the ferulic acid content has shown promising results in several grasses, although these cross-linkages were also proven to exhibit labile behavior during alkaline pretreatment. In general, the release of improved miscanthus varieties will reduce the current bottleneck of efficient biomass conversion for biobased applications and ensure a steady supply of high-quality biomass.

## Figures and Tables

**Table 1:** Different types of pretreatments that have been applied on miscanthus biomass and their effect on biomass composition and enzymatic saccharification. * Simultaneous saccharification and fermentation.

Pretreatment	Crop	Pretreatment Conditions	Extraction Hemicellulose + Lignin	Enzymatic Sacch.	Reference
Organosolv + ball milling	*MxG*	Liquid to solid ratio 10, Ethanol concentration 40%, 170 °C, 120 min	62% lignin removal, 90.6% removal of xylan, mannan, galactan	96.9% cellulose to glucose conversion (6.5% for untreated biomass)	[199]
Bacterial	*M. sac*	Laccase production on M. sac (0.5% *w*/*v*), followed by mixture of enzymes, biomass (4% *w*/*v*), buffer and laccase mediator, 37 °C, 96 h	29.7–59.5% lignin removal, 0.24–0.61% hemicellulose removal	~65.0–87.0% cellulose and ~40.0–78.7% pentose conversion	[232]
Organosolv	*MxG*	Pre-soaked in 500.0 mL water and 40.0 mL of 2 M sulfuric acid, treated with aqueous ethanol (water/ethanol ratio 0.8) + sulfuric acid (0.5%), 170 °C, 60 min	70% lignin removal, 90% xylan and 95% arabinan removal	98% cellulose to glucose conversion	[233]
Ionic liquid	*MxG*	Biomass to solvent ratio 1:5 g/g, Triethylammonium hydrogen sulfate, 180 °C, 15 min	~82% lignin removal, ~90% hemicellulose removal, 1.6% cellulose degradation	~75% cellulose to glucose conversion	[234]
Steam explosion	*M. sac x M. sin*	Pre-soaking + 200 °C, 15 bar, 10 min	~4% lignin removal, 57% hemicellulose removal	~70% cellulose to glucose conversion	[235]
Steam explosion	*M. flo*	175 °C, 20–60 min	42.7% lignin extraction, 70.5% hemicellulose extraction	SSF *, ethanol yield 46.4%	[236]
Fungal	*MxG*	Biomass and inoculum (*MxG* colonized with *Ceriporiopsis subversmipora*, ratio 30–50%), moisture content 60–75%, 28 °C, 28 days	25–35% lignin degradation, 16–24% hemicellulose degradation	35–48% glucose conversion	[237]
Microwave assisted chemical	*MxG*	0.4 M–1.0 M NaOH, 180 °C, 20 min	83.0–94.2% lignin removal, 46.4% hemicellulose removal	150 nmol/mg biomass/h (10 nmol/mg biomass/h for untreated biomass)	[238]

## Data Availability

Not applicable.
